# GnRH Analogues for the Treatment of Endometriosis-Related Pain: A Narrative Review

**DOI:** 10.3390/jcm15145440

**Published:** 2026-07-11

**Authors:** Costin Vlad Anastasiu, Oana Gabriela Dimienescu, Ana-Maria Dull, Ovidiu Dan Grigorescu, Iulia Canditu, Marius Alexandru Moga

**Affiliations:** 1 Department of Medical and Surgical Specialties, Faculty of Medicine, Transilvania University of Brasov, 500019 Brasov, Romania; 2 Regina Maria Hospital, 500091 Brasov, Romania

**Keywords:** endometriosis, pelvic pain, GnRH agonists, GnRH antagonists, elagolix, relugolix, linzagolix, add-back therapy

## Abstract

Endometriosis is a chronic estrogen-dependent condition affecting women of reproductive age and commonly presents with dysmenorrhea, chronic pelvic pain, dyspareunia, and infertility. In a substantial proportion of patients, pain persists despite first-line hormonal treatment, making alternative pharmacologic strategies necessary. GnRH agonists and antagonists act primarily by suppressing ovarian estrogen production and thereby reduce an important hormonal driver of disease activity. Although both classes are effective for pain relief, they differ in onset of action, reversibility, tolerability, and suitability for prolonged use. This narrative review summarizes the current evidence on GnRH-based therapies for endometriosis-associated pain, with particular attention to biological rationale, clinical efficacy, safety, and the role of add-back therapy. Overall, both agonists and antagonists appear to provide clinically significant symptom improvement, especially in women who do not respond adequately to oral contraceptives or progestins. Their clinical use, however, is limited by hypoestrogenic adverse effects such as vasomotor symptoms and bone mineral density loss, which often require monitoring and adjunctive add-back therapy. Oral GnRH antagonists may offer practical advantages in selected patients through rapid action, the absence of a flare effect, and dose-adjustable hormonal suppression, although treatment choice must also consider long-term safety, cost, access, and individual patient characteristics. In summary, GnRH analogues remain useful second-line options, but treatment should be individualized according to symptom severity, previous treatment response, reproductive goals, tolerability, bone health, cost, and availability.

## 1. Introduction

Although endometriosis is widely recognized as an estrogen-dependent disease, its pathophysiology is more complex and likely involves multiple interacting mechanisms beyond ovarian steroid stimulation alone. Genetic susceptibility, progesterone resistance, altered immune function, neuroinflammatory signaling, and broader endocrine–metabolic influences may all contribute to lesion persistence, symptom heterogeneity, and variable treatment response. Recognizing this broader biological context is important when interpreting the benefits and limitations of purely suppressive hormonal strategies. It affects both adolescents and adult women throughout the reproductive years and has an important gynecologic, medical, and socioeconomic impact [1]. Its true prevalence remains difficult to establish because of heterogeneous clinical phenotypes, variable symptom patterns, and the historical dependence on surgical diagnosis. Despite these diagnostic challenges, it is recognized as a common condition worldwide, especially among women with infertility and chronic pelvic pain [2].

The clinical presentation of endometriosis is highly variable and may include dysmenorrhea, non-menstrual pelvic pain, deep dyspareunia, dysuria, fatigue, and reduced fertility. Symptom intensity does not consistently parallel the extent of visible lesions, which makes diagnosis and therapeutic planning more challenging. Many patients also undergo a prolonged diagnostic journey, frequently marked by recurrent symptoms and multiple medical evaluations, with adverse consequences for psychological health, daily functioning, and quality of life [3,4].

The biological mechanisms underlying endometriosis remain only partially understood. Although retrograde menstruation remains the most widely accepted explanatory theory, it does not fully account for the marked variability in lesion distribution, disease phenotype, symptom severity, or treatment response [5]. Other proposed mechanisms, including coelomic metaplasia, embryologic remnants, impaired immune surveillance, genetic susceptibility, progesterone resistance, and abnormal inflammatory signaling, are also likely to contribute to disease onset and progression. Additional endocrine–inflammatory pathways may also deserve attention. Recent observations suggest that aldosterone-related signaling and an increased aldosterone-to-renin ratio may contribute to a proinflammatory internal milieu in endometriosis, although the precise clinical significance of these findings remains to be fully defined. Such observations further support the view that the disease cannot be explained solely by estrogen dependence. Pain in endometriosis is no longer understood as a direct consequence of ectopic implants alone, but rather as the result of interacting inflammatory, neuroangiogenic, peripheral, and central mechanisms [6,7]. Emerging evidence also suggests that broader endocrine–inflammatory pathways may contribute to disease persistence and symptom heterogeneity, further supporting the concept that symptom expression is shaped by a complex biological network rather than by lesion burden alone. Additional endocrine–inflammatory pathways may also deserve consideration. Recent evidence suggests that local renin–angiotensin signaling may participate in the inflammatory milieu of endometriosis, reinforcing the view that the disease cannot be explained solely by ovarian steroid dependence [8]. Current management aims to reduce pain, suppress lesion activity, preserve organ function, and improve quality of life while considering reproductive goals. First-line medical therapy typically includes nonsteroidal anti-inflammatory drugs, combined hormonal contraceptives, and progestin-based regimens. This therapeutic group includes agents such as dienogest and norethindrone acetate, which are commonly used because they may reduce pain while avoiding the profound hypoestrogenism associated with GnRH-based suppression. However, endometriosis management extends beyond these options and may also include depot medroxyprogesterone acetate, the levonorgestrel-releasing intrauterine system, aromatase inhibitors in selected cases, surgery, and multidisciplinary pain management strategies. In this broader therapeutic landscape, GnRH agonists and antagonists should be positioned as second-line options for patients with persistent or refractory symptoms, intolerance to first-line therapy, or complex pain phenotypes [9].

GnRH agonists effectively treat refractory endometriosis-related pain via pituitary desensitization, though their use is often limited by the initial flare effect and hypoestrogenic side effects. More recently, oral GnRH antagonists have emerged as an attractive alternative because they provide rapid, reversible, and dose-dependent suppression of gonadotropin secretion without an initial flare. This pharmacologic profile may offer practical advantages in selected patients, particularly when long-term tolerability and individualized estrogen suppression are clinically relevant [10,11]. In this context, GnRH agonists and GnRH antagonists can be compared as therapeutic options for endometriosis-associated pain, with attention to mechanisms of action, evidence from major clinical trials, safety profiles, and their place in individualized treatment strategies [12]. This manuscript was developed as a clinically oriented narrative review. A structured literature search was conducted using PubMed and Google Scholar to identify relevant publications on endometriosis-associated pain, GnRH agonists, GnRH antagonists, add-back therapy, safety considerations, and long-term therapeutic positioning. Search terms included combinations of “endometriosis”, “pelvic pain”, “dysmenorrhea”, “GnRH agonist”, “GnRH antagonist”, “elagolix”, “relugolix”, “linzagolix”, “leuprolide”, “goserelin”, “nafarelin”, “add-back therapy”, “bone mineral density”, “fertility”, and “central sensitization”. We included randomized clinical trials, comparative studies, international guidelines and recent narrative or systematic reviews with direct relevance to pain outcomes, tolerability, and clinical use. This review was designed as a narrative rather than systematic synthesis; therefore, the selection of references was purposeful and clinically focused rather than exhaustive, and no formal PRISMA-based screening or structured risk-of-bias assessment was performed.

## 2. Pathophysiology of Pain in Endometriosis

Pain in endometriosis is a complex, multidimensional clinical entity that does not consistently correlate with laparoscopic staging or lesion burden. Marked symptoms may occur in women with limited visible disease, whereas some patients with extensive lesions report comparatively less discomfort [13,14,15]. This apparent mismatch suggests that pain is shaped by the combined influence of local inflammation, peripheral sensitization, altered central pain processing, and coexisting pain conditions. For this reason, endometriosis-associated pain should be understood as more than a purely anatomical consequence of ectopic tissue [16,17,18]. The mechanistic evidence discussed in this review is derived from different levels of investigation, including human clinical observations, analyses of eutopic and ectopic endometrial tissue, peritoneal fluid studies, and experimental data from in vitro and animal models. This distinction is important because the biological plausibility of inflammatory, neoangiogenic, and neuroimmune pathways is supported by a translational body of evidence rather than by a single type of study.

### 2.1. Inflammation

Endometriosis is widely regarded as a chronic inflammatory condition in which persistent immune dysregulation contributes both to lesion development and to pain generation [18,19]. Ectopic endometrium-like tissue interacts with the peritoneal cavity in a manner that creates a biologically active microenvironment rich in inflammatory cells, cytokines, chemokines, growth factors, and oxidative mediators. This inflammatory setting is not merely a secondary consequence of ectopic implantation; instead, it supports lesion persistence, vascular development, and nociceptive signaling [20]. In normal conditions, menstrual material that reaches the peritoneal cavity is usually removed by innate immune clearance mechanisms. In patients with endometriosis, this process seems to be less effective, which allows endometrial cells to remain in place and interact with the local immune environment. Repeated bleeding from ectopic lesions further enhances tissue injury through iron release, reactive oxygen species, and damage-associated molecular signals, thereby sustaining oxidative stress and inflammation. This self-perpetuating cycle contributes to disease chronicity and may help explain why pain can persist even when the visible extent of lesions is limited [19,20,21].

Among immune cells, macrophages appear to have a central role in the inflammatory architecture of endometriosis. Current evidence suggests that macrophage behavior in endometriosis is better understood as a spectrum of context-dependent activation states rather than a strict M1/M2 dichotomy [21]. Nevertheless, both pro-inflammatory and immunoregulatory macrophage subsets seem to participate in disease maintenance [22]. Through the release of mediators such as IL-1β, IL-6, TNF-α, MCP-1, and TGF-β, macrophages support local inflammation, extracellular matrix remodeling, angiogenesis, fibrosis, and neuroimmune crosstalk [7,23]. At the same time, altered phagocytic function may reduce the clearance of ectopic endometrial material, thereby favoring lesion persistence. Other immune cell populations further intensify this inflammatory environment. Activated mast cells, which are particularly abundant in deeply infiltrating lesions, release histamine, tryptase, serotonin, leukotrienes, and pro-inflammatory cytokines that can sensitize peripheral nerve endings and contribute to pain amplification [13,24]. Neutrophils also appear to participate, especially in earlier phases of lesion development, by producing pro-angiogenic factors such as vascular endothelial growth factor (VEGF) and facilitating the establishment of ectopic implants [25]. These immune–vascular interactions are highly relevant because inflammation and neuroangiogenesis often evolve in parallel in symptomatic disease. A critical component of this inflammatory signaling is the overexpression of cyclooxygenase-2 (COX-2), resulting in increased prostaglandin E2 (PGE2) production [25,26]. This pathway is stimulated by inflammatory cytokines, estrogenic signaling, and local hypoxic stress. PGE2 has multiple downstream effects that are directly relevant to pain: it enhances uterine contractility, lowers nociceptor activation thresholds, promotes aromatase expression and local estrogen biosynthesis, and thereby contributes to a positive feedback loop between inflammation and estrogen dependence. In this way, inflammatory mediators do not merely accompany the disease; they help biologically sustain the hormonal and nociceptive conditions that drive endometriosis-associated pain [7,20]. In addition to the more frequently discussed inflammatory mediators, emerging evidence suggests that other endocrine–inflammatory pathways may also contribute to the disease microenvironment. In this context, aldosterone-related signaling has been proposed as a potential contributor to inflammatory activation in endometriosis, although its precise clinical significance remains less clearly established than that of the better-characterized estrogen-dependent and cytokine-mediated pathways. Table 1 summarizes the inflammatory mediators implicated in endometriosis.

Taken together, these findings indicate that inflammation in endometriosis is not a single isolated event, but a coordinated process involving defective immune clearance, chronic cytokine signaling, oxidative stress, and reciprocal interactions between immune cells, ectopic tissue, and peripheral nerves. GnRH analogues may contribute to pain reduction partly through suppression of estrogen-dependent inflammatory signaling and downstream neuroangiogenic activity. However, although this mechanism is biologically plausible and supported by translational evidence, direct clinical evidence in humans demonstrating a specific anti-inflammatory effect at the lesion level remains limited.

### 2.2. Peripheral Sensitization

Peripheral sensitization is a major contributor to endometriosis-associated pain and arises when persistent exposure to inflammatory and neuroactive mediators lowers the activation threshold of peripheral nociceptors. In endometriosis, ectopic lesions are not inert tissue implants; rather, they function as biologically active sites that continuously interact with immune cells, blood vessels, and sensory nerve endings. This local environment promotes exaggerated nociceptive signaling and helps explain why pain may persist or intensify independently of lesion size alone [14,33]. The peritoneal and lesional microenvironment in endometriosis is enriched in pro-inflammatory cytokines and chemokines, including interleukins, tumor necrosis factor-α, and prostaglandin-related mediators [29,35]. These molecules directly affect sensory neurons and indirectly amplify nociception by recruiting and activating macrophages, mast cells, and other immune cells. In parallel, lesion-associated oxidative stress and recurrent microhemorrhage further sustain tissue irritation and inflammatory signaling. Over time, this persistent biochemical stimulation favors a state in which peripheral nerve endings become increasingly responsive to stimuli that would otherwise be less painful or even innocuous [19,35,36].

An important feature of peripheral sensitization in endometriosis is the close relationship between inflammation and neuroangiogenesis [37,38]. Endometriotic lesions produce angiogenic and neurotrophic factors, particularly vascular endothelial growth factors (VEGF) and nerve growth factors (NGF), which promote both vascular support and sensory nerve ingrowth [32,33]. Newly formed nerve fibers, especially small-diameter sensory fibers, increase the capacity of lesions to generate and transmit pain signals. In this setting, lesion progression is accompanied not only by inflammation, but also by the establishment of a nerve-rich microenvironment capable of maintaining chronic nociceptive input [28].

Peripheral sensitization is also reinforced by changes in receptor and ion-channel expression on sensory fibers. Of particular interest is transient receptor potential vanilloid 1 (TRPV1) [34], a nociceptive channel involved in thermal, chemical, and inflammatory pain signaling. Increased TRPV1 expression has been associated with heightened pain responsiveness in endometriosis and may contribute to the transition from cyclical nociception to persistent pelvic pain [35]. Neuroimmune interactions further intensify this process, as mast-cell mediators such as histamine and NGF, together with macrophage-derived inflammatory signals, can enhance neuronal excitability and sustain local hyperalgesia [36,37,38,39]. Another relevant mechanism is the intimate anatomical relationship between endometriotic lesions and peripheral nerves. Deep infiltrating disease has been associated with nerve-fiber remodeling and perineural involvement, suggesting that the lesion itself may become integrated into local pain pathways [19,22,36]. Macrophages can accumulate near nerve fibers and release mediators that support axonal growth and sensitization, while lesion-derived factors attract additional immune cells and reinforce this feed-forward loop [22,39]. Thus, peripheral sensitization in endometriosis should be understood as the consequence of a sustained neuroinflammatory dialogue between ectopic tissue, immune cells, and sensory nerves.

This framework is clinically relevant because therapies that suppress ovarian steroid production may also reduce peripheral sensitization indirectly by attenuating estrogen-dependent inflammatory and neuroangiogenic activity. However, although this mechanism is biologically plausible, direct clinical evidence in humans demonstrating a specific anti-inflammatory effect at the lesion level remains limited. These mechanisms indicate that peripheral pain generation in endometriosis extends beyond simple inflammatory irritation.

### 2.3. Central Sensitization and Cross Sensitization

While peripheral nociceptive input is essential for the initiation of pain in endometriosis, persistent symptoms increasingly appear to involve central sensitization, a process in which the central nervous system becomes more responsive to repeated or sustained pain signaling. In this state, pain is no longer determined solely by ongoing peripheral lesion activity; instead, spinal and supraspinal pathways become hyperexcitable, resulting in pain amplification, reduced pain thresholds, temporal summation, and pain that may extend beyond the original site of pelvic pathology [2,15,40,41]. This concept helps explain why some patients continue to report severe pain despite apparently limited disease burden or after local treatment of visible lesions.

Central sensitization develops when repeated nociceptive traffic from endometriotic lesions induces maladaptive plasticity in the spinal cord, dorsal root ganglia, and higher pain-processing centers [14,33]. Broader endocrine–metabolic influences may also contribute to disease heterogeneity. Possible androgen-related mechanisms and the recognized clinical overlap between endometriosis and certain endocrine–metabolic conditions, including polycystic ovary syndrome phenotypes, deserve acknowledgment as part of the wider biological context of chronic pelvic pain, even though these pathways remain less clearly defined in relation to GnRH-based treatment response. Under these conditions, second-order neurons may become hyperexcitable, descending inhibitory pathways may function less effectively, and sensory processing may shift toward pain facilitation rather than pain containment. As a result, patients may experience pain amplification, reduced pain thresholds, temporal summation, and pain extending beyond the original site of pelvic pathology [33]. These mechanisms are particularly relevant in women with chronic pelvic pain, dyspareunia, and mixed nociceptive–neuropathic symptom profiles. Emerging neurobiological evidence also supports the concept that endometriosis-associated pain involves changes at the level of the brain. Functional and structural imaging studies cited in literature have suggested alterations in regions involved in pain perception, emotional appraisal, and descending modulation [30,32]. Although such findings are not specific to endometriosis, they are consistent with a chronic pain phenotype characterized by enhanced central responsiveness and impaired endogenous pain control [17]. In this context, pain becomes a multidimensional phenomenon shaped not only by lesion biology, but also by central processing, stress regulation, and affective modulation.

Neuroinflammation appears to be one of the key bridges between persistent peripheral inflammation and central pain amplification. Cytokines, prostaglandins, neurotrophic factors, and neuropeptides generated in the lesional environment can influence dorsal root ganglion neurons and central nociceptive circuits. Mediators such as NGF, TNF-α, IL-1β, IL-6, and PGE2 are of particular interest because they participate in both local inflammation and neuronal sensitization [19,27,31,32]. Once these signals repeatedly reach the central nervous system, they may promote long-term excitability changes that sustain pain even when peripheral stimuli fluctuate over time.

Central sensitization may also interact with broader neuroendocrine and psychosocial factors [31]. Chronic pain in endometriosis is frequently accompanied by fatigue, sleep disturbance, anxiety, depressive symptoms, and pain catastrophizing, all of which may further intensify central pain processing [14]. Moreover, dysregulation of stress-response pathways may reduce the efficiency of endogenous analgesic mechanisms, thereby reinforcing the persistence of pain. Broader endocrine–metabolic influences may also contribute to disease heterogeneity. In particular, possible androgen-related mechanisms and the recognized clinical overlap between endometriosis and certain endocrine–metabolic conditions, including polycystic ovary syndrome phenotypes, deserve acknowledgment as part of the wider biological context of chronic pelvic pain, even though these pathways remain less clearly defined in relation to GnRH-based treatment response. For this reason, central sensitization should not be viewed as a purely neurologic phenomenon, but rather as part of a complex biopsychosocial pain model in which inflammation, hormonal signaling, emotional distress, and chronic nociceptive exposure converge. From a clinical perspective, recognizing central sensitization is important because it may influence treatment response. Patients with a prominent centralized pain component may derive incomplete benefit from lesion-directed strategies alone and may require broader multimodal management. In endometriosis, this reinforces the need for individualized care that addresses both the peripheral generators of pain and the central mechanisms responsible for pain persistence and symptom amplification [14,15,16,31,41]. This mechanistic framework may also help explain why the clinical response to GnRH-based therapy is variable across patients. By suppressing ovarian estrogen production, GnRH analogues may reduce the peripheral nociceptive input generated by active lesions, inflammatory mediators, and neuroangiogenic signaling. However, once central sensitization and pain amplification are established, endocrine suppression alone may be insufficient to fully reverse chronic pain processing. In such cases, hormonal therapy should be understood as one component of a broader multimodal approach rather than a complete solution to persistent pain. In this context, the potential contribution of GnRH analogues to pain relief should be interpreted mainly through their effect on the peripheral hormonal and inflammatory drive that sustains nociceptive input. By reducing estrogen-dependent lesion activity and the associated peripheral sensitizing environment, these therapies may indirectly lessen the afferent input that contributes to the maintenance of central sensitization. However, they do not directly reverse all established central pain-amplification mechanisms, which helps explain why some patients continue to experience pain despite adequate hormonal suppression and why multimodal management remains necessary [42].

In this context, the potential contribution of GnRH analogues to pain relief should be interpreted mainly through their effect on the peripheral hormonal and inflammatory drive that sustains nociceptive input. By reducing estrogen-dependent lesion activity and the associated peripheral sensitizing environment, these therapies may indirectly lessen the afferent input that contributes to the maintenance of central sensitization. However, they do not directly reverse all established central pain-amplification mechanisms, which helps explain why some patients continue to experience pain despite adequate hormonal suppression and why multimodal management remains necessary [42].

## 3. GnRH Analogues

**Figure 1:**
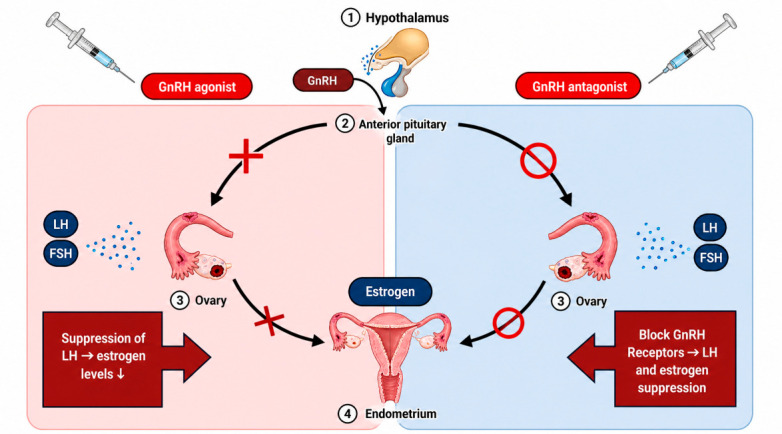
Main mechanisms of action of GnRH analogues in endometriosis-associated pain.

GnRH analogues are generally used as second-line therapies for endometriosis-associated pain, particularly when first-line medical treatment with oral contraceptives or progestogens is ineffective or poorly tolerated. The clinical management of endometriosis aims to reduce pain, suppress lesion activity, preserve organ function when possible, and improve quality of life while taking reproductive goals into account. First-line treatment generally includes non-steroidal anti-inflammatory drugs, combined hormonal contraceptives, and progestin-based therapies. However, a substantial subgroup of patients does not achieve adequate symptom control with these approaches, creating a need for second-line options. In this setting, GnRH agonists and GnRH antagonists remain clinically important therapies whose mechanisms of action, efficacy, safety profiles, and role in individualized treatment deserve careful evaluation [43,44]. Progesterone receptor insensitivity, often described clinically as progesterone resistance, is considered an important contributor to treatment failure and persistent pain in endometriosis. This concept also helps explain why synthetic progestins that act through progesterone receptors, such as dienogest and norethindrone acetate, remain clinically important in the management of endometriosis-associated pain before escalation to GnRH-based suppression. In many patients, these less suppressive options may provide meaningful symptom relief while avoiding some of the hypoestrogenic adverse effects associated with GnRH analogues. In such situations, both GnRH agonists and GnRH antagonists represent relevant alternatives. Figure 1 illustrates the main mechanisms through which GnRH analogues exert their effects.

### 3.1. GnRH Agonists

GnRH agonists are established second-line therapies for endometriosis-associated pain, particularly in women with moderate-to-severe symptoms who do not achieve adequate symptom control with combined hormonal contraceptives or progestins [43]. Their pharmacological action is characterized by an initial transient stimulation of pituitary GnRH receptors, followed by receptor downregulation and suppression of gonadotropin secretion with subsequent ovarian estrogen deprivation. This hypoestrogenic state reduces hormonal support for endometriotic lesions, contributes to lesion regression, attenuates inflammatory activity, and may reduce pain transmission driven by persistent peripheral nociceptive input [45,46,47,48]. Clinical trials involving agents such as leuprolide, goserelin, and nafarelin have demonstrated meaningful improvement in dysmenorrhea, chronic pelvic pain, and dyspareunia. However, prolonged estrogen suppression is associated with adverse effects including vasomotor symptoms, sleep disturbance, vaginal dryness, reduced libido, mood changes, and bone mineral density loss. For this reason, treatment duration is generally limited in the absence of add-back therapy, which is now commonly used to improve tolerability and support longer treatment courses [44,45,46,47,48].

Add-back therapy is a key strategy for improving the tolerability of GnRH agonist treatment and reducing the consequences of prolonged hypoestrogenism, particularly bone mineral density loss and vasomotor symptoms. Its aim is to provide sufficient hormonal support to mitigate treatment-related adverse effects without reactivating endometriosis-associated symptoms. One of the best-known approaches uses norethindrone acetate, although other combined add-back strategies have also been evaluated. Importantly, this approach should be distinguished from fixed-dose oral combination regimens used with certain GnRH antagonists [49,50,51,52].

Clinical benefit has been demonstrated in older randomized trials involving nafarelin, leuprolide, and goserelin, with efficacy broadly comparable to danazol and superior to placebo for several pain outcomes [53,54,55,56]. However, these studies were heterogeneous in design, often emphasized laparoscopic lesion regression, and did not consistently use standardized pain-response criteria or patient-reported quality-of-life endpoints. This distinction is important because evidence derived from GnRH agonist monotherapy should not be conflated with data specifically obtained from add-back therapy studies.

A major limitation of GnRH agonists is the adverse-effect profile associated with estrogen deprivation, including hot flushes, vaginal dryness, mood changes, sleep disturbance, reduced libido, and bone mineral density loss [44,51]. For this reason, add-back therapy has become a key component of prolonged treatment. Earlier studies with leuprolide showed that norethindrone acetate alone or combined with low-dose estrogen preserved bone mineral density without compromising pain relief, thereby improving the feasibility of longer treatment courses [57,58]. This distinction is important because evidence derived from GnRH agonist monotherapy should not be conflated with data specifically obtained from add-back therapy studies.

Representative clinical studies evaluating GnRH agonists in endometriosis-associated pain are summarized in Table 2. Although large new randomized efficacy trials of GnRH agonists have been scarce after 2020, recent bibliography confirms that this class remains clinically important, while emphasizing that contemporary research has shifted toward long-term safety, treatment extension, and comparative therapeutic positioning rather than novel efficacy trials. For clarity, safety findings and interpretative clinical comments were separated in the revised table to improve consistency and comparability across studies.

### 3.2. GnRH Antagonists

Like GnRH agonists, antagonists are also associated with hypoestrogenic symptoms, especially at higher doses or during prolonged administration. Vasomotor symptoms, headache, and bone mineral density reduction remain important limitations and should be considered during treatment selection and follow-up [10,50].

#### 3.2.1. Elagolix

Elagolix is among the best-studied oral GnRH antagonists for endometriosis-related pain. Data from the ELARIS (pivotal Phase III clinical trials evaluating the efficacy of elagolix) showed improvement in both dysmenorrhea and non-menstrual pelvic pain compared with placebo, supporting its use in women with moderate-to-severe symptoms. Oral administration and rapid pharmacodynamic action make this drug practical in routine care. Nevertheless, clinical benefits must be weighed against tolerability, since higher doses are usually associated with greater symptom control but also with more evident hypoestrogenic adverse effects, including hot flushes and bone mineral density decline [68,69,70,71,72].

#### 3.2.2. Relugolix

Relugolix has been studied mainly as combination therapy with estradiol and norethindrone acetate in the SPIRIT trials. This strategy is clinically important because it seeks to maintain the analgesic effect of ovarian suppression while reducing vasomotor symptoms and bone loss. Current evidence indicates that relugolix combination therapy improves endometriosis-associated pain and may represent a more sustainable therapeutic approach for longer-term management. This therapeutic model should be distinguished from traditional add-back therapy used with GnRH agonists. In the agonist setting, add-back therapy is introduced as an adjunctive strategy to reduce the consequences of prolonged hypoestrogenism while preserving pain control. By contrast, relugolix combination therapy is designed as a fixed-dose integrated regimen in which estrogen and progestin are incorporated from the outset to balance efficacy and tolerability. This distinction has practical implications for counselling, monitoring, and interpretation of safety outcomes during longer-term treatment. In practical terms, this regimen illustrates the shift in endometriosis treatment toward individualized hormonal suppression instead of profound estrogen deprivation alone. The balance between efficacy, tolerability, and patient acceptability is central to its clinical value [73,74,75,76,77,78].

#### 3.2.3. Linzagolix

Linzagolix is another oral GnRH antagonist that has shown encouraging results in endometriosis-related pain [79,80]. Because its effect is dose-dependent, it may allow different therapeutic strategies based on symptom severity and tolerability. Lower-dose regimens may improve symptoms with a smaller hypoestrogenic burden, whereas higher doses may require add-back therapy to optimize safety. This flexibility may be useful in patients whose therapeutic needs differ according to age, pain pattern, previous treatment exposure, and reproductive plans. As with the other drugs in this class, however, long-term bone safety remains an important concern [79,80,81,82]. Treatment duration with oral GnRH antagonists also requires careful individualization. Although these agents offer practical advantages such as rapid onset and dose-dependent suppression, duration limits are influenced by dose, the use of combination or add-back regimens, bone health considerations, regulatory approvals, and the patient’s overall risk profile. For this reason, longer use should be interpreted within the context of specific regimen design rather than assumed for the entire drug class. This distinction is clinically important. Traditional add-back therapy with GnRH agonists is typically introduced as a strategy to improve tolerability during ongoing ovarian suppression, whereas fixed-dose oral combination regimens with GnRH antagonists are designed from the outset to combine suppression with hormonal protection. Although both approaches aim to reduce hypoestrogenic toxicity, their pharmacologic rationale, implementation, and monitoring considerations are not identical. Table 3 summarizes clinical trials evaluating oral GnRH antagonists in endometriosis-associated pain.

The available evidence should also be interpreted in light of important differences between older studies of GnRH agonists and more recent trials of oral GnRH antagonists. Earlier agonist trials were often smaller, methodologically heterogeneous, and conducted before the widespread use of standardized responder definitions, validated patient-reported outcome measures, and contemporary safety monitoring frameworks. By contrast, the major antagonist programs used more structured pain endpoints, quality-of-life instruments, and predefined safety assessments. These differences limit direct cross-trial comparisons and suggest that apparent advantages of newer regimens may reflect, at least in part, differences in trial design and outcome reporting rather than pharmacologic superiority alone. Recent advances in the medical treatment of endometriosis-associated pain have centered mainly on oral GnRH antagonists and regimens combined with add-back therapy. In comparison with earlier studies of GnRH agonists, these more recent trials more often incorporate standardized responder criteria, patient-reported outcome measures, and longer-term assessments of bone safety [10,50,71]. The available evidence should also be interpreted considering important methodological differences between older studies of GnRH agonists and more recent trials of oral GnRH antagonists. Earlier agonist studies were often smaller, methodologically heterogeneous, and conducted before the widespread use of standardized responder definitions, validated patient-reported outcome measures, and contemporary safety-monitoring frameworks. By contrast, the major antagonist programs used more structured pain endpoints, quality-of-life instruments, and predefined safety assessments. These differences limit direct cross-trial comparisons and suggest that some apparent advantages of newer regimens may reflect, at least in part, differences in trial design and outcome reporting rather than pharmacologic superiority alone [83].

The available evidence for GnRH agonists and antagonists comes from different phases of endometriosis research. Earlier trials of GnRH agonists showed clear efficacy, but many were conducted before standardized pain-response criteria, validated quality-of-life tools, and structured long-term safety assessments became routine. In contrast, more recent studies of oral GnRH antagonists have generally used endpoints that are closer to current clinical expectations and are therefore easier to interpret in modern patient-centered practice.

Safety assessment remains central to treatment selection and follow-up. In addition to vasomotor symptoms, vaginal dryness, sleep disturbance, reduced libido, and bone mineral density loss, clinicians should consider the potential impact of prolonged hypoestrogenism on long-term bone health and quality of life. Baseline clinical assessment should include attention to fracture risk, menstrual and reproductive history, and the anticipated duration of therapy. In patients receiving oral antagonist-based combination regimens, the estrogen–progestin component also requires attention to thromboembolic and cardiovascular risk, as well as contraindications to estrogen exposure. Psychiatric history is also relevant, particularly when considering therapies associated with mood changes, depression, or possible suicidal ideation. Pregnancy must be excluded before treatment initiation, and counseling regarding contraception is essential because these therapies are not appropriate during active attempts to conceive.

In clinical practice, GnRH agonists continue to be useful second-line treatments, especially for patients accustomed to injectable therapy or in settings where newer oral agents are less accessible [84,85]. Safety assessment remains central to treatment selection and follow-up. In addition to vasomotor symptoms, vaginal dryness, sleep disturbance, reduced libido, and bone mineral density loss, clinicians should consider the impact of prolonged hypoestrogenism on long-term bone health and quality of life. Baseline evaluation should include attention to fracture risk, menstrual and reproductive history, and the anticipated duration of therapy. Psychiatric history is also relevant, particularly when considering therapies associated with mood changes, depression, or possible suicidal ideation, as reported with elagolix. Pregnancy should be excluded before treatment initiation, and contraception counselling is essential because these therapies are not appropriate during active attempts to conceive. In patients receiving antagonist-based combination regimens containing estrogen and progestin, thromboembolic and cardiovascular risk, as well as contraindications to estrogen exposure, should also be considered. Hepatic considerations may also be relevant depending on the regimen used and the patient’s clinical background. However, the initial flare phenomenon, reduced pharmacologic flexibility, and hypoestrogenic adverse effects may limit prolonged use unless add-back therapy is included [57,58,74]. Earlier add-back studies with leuprolide demonstrated that symptom control could be maintained while preserving bone health, an observation that remains highly relevant today [66].

Recent advances in the medical treatment of endometriosis-associated pain have centered mainly on oral GnRH antagonists and regimens combined with add-back therapy. Combination therapy and newer elagolix- or linzagolix-based regimens have demonstrated clinically meaningful reductions in dysmenorrhea and non-menstrual pelvic pain, alongside more structured data on bone mineral density, functional status, and quality of life [73,74,81,82,83]. The clinical role of GnRH analogues should also be interpreted within the broader therapeutic landscape of endometriosis. First-line and alternative medical options include combined hormonal contraceptives, progestins such as dienogest and norethindrone acetate, depot medroxyprogesterone acetate, and the levonorgestrel-releasing intrauterine system. In selected refractory cases, aromatase inhibitors may also be considered, usually in combination with other suppressive strategies. In addition, surgery and multidisciplinary pain management remain essential components of care in many patients. Without this broader context, the position of GnRH analogues as second-line therapies cannot be adequately defined.

Hormonal suppression alone is not adequate for every woman with endometriosis-associated pain. In patients with central sensitization, pelvic floor dysfunction, bowel symptoms, or bladder pain, persistent symptoms may be driven by mechanisms that are not limited to ovarian steroid dependence [14,33,42,86]. For this reason, GnRH-based therapy should be incorporated into a broader multimodal management plan that may also include analgesic optimization, pelvic floor physiotherapy, psychological support, and surgery when indicated. It is also important to emphasize that chronic pain in endometriosis may often be approached initially or concomitantly with less invasive therapeutic strategies, including analgesic optimization, first-line hormonal treatments, and progestin-based regimens. In this context, GnRH analogues should be viewed as important second-line options rather than as obligatory early interventions in all patients. Fertility and reproductive planning should also play a central role in treatment selection. Although hormonal suppression may reduce pain, it is not appropriate for women actively trying to conceive because these therapies suppress ovulation and do not directly improve fertility. In such cases, clinical management should be coordinated with reproductive goals and may require integration with surgery or assisted reproductive strategies when indicated. In addition, the presence of central sensitization and overlapping pain syndromes reinforces the need for multidisciplinary care, including pelvic floor physiotherapy, psychological support, and individualized management of bowel, bladder, and musculoskeletal pain contributors.

Safety assessment remains central to treatment selection and follow-up. In addition to vasomotor symptoms, vaginal dryness, sleep disturbance, reduced libido, and bone mineral density loss, clinicians should consider psychiatric history, contraception needs, pregnancy exclusion before treatment initiation, and the thromboembolic or cardiovascular risk associated with estrogen-containing combination regimens. Treatment decisions should therefore be based on shared decision-making and should consider efficacy, bone health, reproductive plans, tolerability, psychiatric profile, cost, and availability [86]. Consequently, treatment decisions should be based on shared decision-making and should consider individual efficacy, adverse effects, treatment duration, bone health, reproductive plans, cost, and availability. Cost, access, and route of administration also influence real-world treatment selection. Depot GnRH agonists remain clinically relevant in many settings because of long-standing familiarity and, in some healthcare systems, greater availability. Oral GnRH antagonists may offer practical advantages related to rapid onset, reversibility, and avoidance of the flare effect, but these potential benefits must be weighed against cost, local access, reimbursement status, and the need for individualized safety assessment. Accordingly, the relative value of each option may differ substantially across clinical settings.

Several subgroup considerations deserve emphasis. GnRH-based suppression is not an appropriate active treatment strategy for women who are currently trying to conceive, and its use should be integrated into a broader reproductive plan. In adolescents, treatment decisions require caution because of concerns related to bone accrual, long-term tolerability, and the need to avoid unnecessary prolonged hypoestrogenism during a critical developmental period. Additional caution is warranted in patients with low baseline bone mineral density, a history of mood disorders, or previous suicidal ideation. These factors reinforce the need for individualized treatment selection rather than a uniform preference for any single GnRH-based regimen.

The available evidence must also be interpreted considering important methodological differences between older GnRH agonist studies and more recent antagonist trials. Earlier agonist studies were often smaller and more heterogeneous, whereas modern antagonist programs have generally used more standardized responder definitions, patient-reported outcomes, and structured safety assessment. These differences limit direct cross-trial comparison and suggest that some apparent advantages of newer regimens may also reflect differences in trial design and reporting standards rather than pharmacologic superiority alone.

Taken together, these considerations support a stepped and individualized therapeutic approach. Although GnRH analogues remain highly relevant in patients with refractory or moderate-to-severe pain, many patients may initially benefit from less suppressive strategies, particularly progestin-based regimens and other less invasive options that may provide symptom relief with a lower burden of menopausal-like adverse effects.

The authors declare that GenAI was used in the creation of this manuscript.

## 4. Conclusions

GnRH agonists and antagonists remain important second-line options for the management of endometriosis-associated pain in patients who do not achieve adequate symptom control with first-line therapies. GnRH agonists continue to have an established role, particularly when used with appropriate add-back therapy and monitoring, whereas oral GnRH antagonists offer practical advantages such as rapid onset of action, oral administration, and dose-dependent suppression. However, these characteristics should not be interpreted as evidence of universal superiority. For many women, chronic pain may also be managed with less invasive medical options, including progestin-based therapies and other individualized multimodal strategies, before escalation to GnRH-based suppression is required. Treatment selection should therefore remain individualized and multidisciplinary, taking into account symptom severity, reproductive goals, long-term tolerability, bone health, psychiatric history, cost, treatment availability, and the broader biological complexity of the disease. Although the present manuscript is intended as a narrative clinical review rather than a formal treatment guideline, the therapeutic principles discussed may support a stepwise and individualized approach to treatment selection in routine practice.

## Figures and Tables

**Table 1:** Key inflammatory mediators and pathways involved in the pathophysiology of endometriosis-associated pain.

Biomarker	Pathophysiologic Role in Endometriosis	Association with Pain
IL-1 β [27]	Central pro-inflammatory cytokine involved in lesion-related immune activation; promotes inflammatory amplification, macrophage recruitment, and downstream mediator release, including COX-2-related signaling.	Contributes to nociceptor sensitization and sustains the inflammatory microenvironment associated with dysmenorrhea and chronic pelvic pain.
IL-6 [28]	Supports stromal-cell proliferation, immune dysregulation, oxidative stress, and chronic inflammatory persistence; fibrotic remodeling and JAK/STAT-related inflammatory signaling.	Elevated in inflammatory disease states and linked to chronic pelvic pain biology, although direct clinical discrimination by pain phenotype remains inconsistent across sample types.
TNF-α [27,29]	Major upstream inflammatory cytokine that amplifies lesion inflammation, promotes immune-cell activation, interacts with neuroinflammatory pathways.	Involved in peripheral sensitization and in pathways that enhance NGF synthesis and pain transmission.
IL-8 (CXCL8) [23]	Chemotactic and pro-angiogenic cytokine involved in leukocyte recruitment, lesion maintenance, and persistence of the inflammatory peritoneal milieu.	Linked to hyperalgesia and neuroimmune activation; mast-cell and macrophage–rich lesions may enhance its nociceptive relevance.
IL-17 [24]	Produced mainly in Th17-skewed inflammatory responses; enhances secretion of pro-inflammatory and angiogenic mediators contributes to immune imbalance.	Supports a pro-nociceptive microenvironment through inflammatory amplification rather than being a standalone pain marker.
CCL2/MCP-1 [14,15]	Chemokine involved in monocyte/macrophage recruitment to lesions and to nerve-associated microenvironments; contributes to chronic immune-cell trafficking and lesion persistence.	Supports neuroimmune crosstalk by facilitating macrophage accumulation near nerve fibers, thereby contributing to sensitization.
COX-2/PGE2 axis [29,30]	COX-2 overexpression drives PGE2 production, which sustains lesion viability, aromatase activity, estrogen-dependent feedback, and uterine hypercontractility.	PGE2 lowers nociceptive thresholds, promotes dysmenorrhea, and is one of the clearest mediators linking inflammation to pain.
VEGF/VEGF-A [28]	Key angiogenic mediator promoting endothelial proliferation, vascular permeability, lesion vascularization, and survival; produced by stromal cells and immune cells including macrophages and neutrophils.	Supports lesion innervation/vascularization and correlates with biologically active disease, thereby facilitating pain persistence.
TGF-β1 [31]	Contributes to fibrosis, fibroblast activation, extracellular matrix remodeling, and immunologic dysregulation; enhances prostaglandin-related inflammatory signaling.	Particularly relevant for chronic pain and deep disease phenotypes where fibrosis and persistent inflammation coexist.
NGF [32,33]	Neurotrophic factor involved in sensory nerve sprouting, hyperinnervation, and neurogenic inflammation; produced in part by macrophages and mast cells within lesion-associated inflammatory niches.	One of the most important mediators linking inflammation to peripheral sensitization and endometriosis-associated pain.
Histamine [34]	Mast-cell–derived mediator involved in neuroimmune signaling, vasodilation, interaction with nociceptive pathways; may further stimulate NGF-related signaling.	Contributes to nociceptor sensitization and mast-cell–driven pain amplification, especially in neuroinflammatory models of endometriosis.

**Table 2:** Representative clinical studies of GnRH agonists in endometriosis-associated pain.

Therapeutic Subgroup	Ref	GnRH Agonist Regimen	Comparator	Efficacy Findings	Safety Outcomes	Clinical Comments
Nafarelin	[51]	Nafarelin 400 or 800 μg/day, intranasal	Danazol 800 mg/day, oral	Mean laparoscopic scores declined in all groups; >80% of patients in each arm had reduced disease extent.	Hypoestrogenic adverse effects were more typical of nafarelin treatment.	Overall efficacy was comparable across groups.
	[52]	Nafarelin 400 μg/day, intranasal	Danazol 600 mg/day, oral	Complete regression in 30% and partial regression in 57%; both treatments reduced active disease without a significant difference in efficacy.	Hot flushes and headaches were more frequent with nafarelin, whereas weight gain was more common with danazol.	Head-to-head comparative trial.
	[53]	Nafarelin 200 μg BID, intranasal	Danazol 200 mg BID, oral	AFS endometriosis scores and symptom severity scores decreased in both groups, indicating similar efficacy.	No major between-group differences in overall efficacy; tolerability profile differed according to mechanism of action.	Concise head-to-head efficacy comparison.
	[55]	Nafarelin 200 μg BID, intranasal	Danazol	Clinical improvement occurred in 94% of the nafarelin group and 91% of the danazol group; resolution of physical findings was comparable.	Hypoestrogenic symptoms were associated with nafarelin, whereas androgenic effects were more typical of danazol.	Symptom-oriented companion study.
	[56]	Nafarelin 200 μg BID, intranasal	Danazol 200 mg TID, oral	Disease extent and symptoms improved in both groups; symptoms remained less severe than baseline after discontinuation.	Overall tolerability was acceptable in both groups, with adverse effects reflecting the endocrine mechanism of each drug.	Comparable effectiveness during treatment and follow-up.
Leuprolide	[57]	Leuprolide acetate depot 3.75 mg monthly, IM	Placebo	Greater improvement in dysmenorrhea, pelvic pain, and pelvic tenderness than placebo.	Menstrual suppression and menopausal-range estradiol levels were achieved; vasomotor symptoms were reported more frequently.	Placebo-controlled efficacy study.
	[58]	Leuprolide acetate depot 3.75 mg monthly	Danazol 800 mg/day	Both drugs were associated with frequent but largely reversible adverse effects.	Bone density loss was more pronounced with leuprolide; danazol had a less favorable lipid/metabolic profile.	Safety comparison is clinically important.
	[59]	Leuprolide acetate depot 3.75 mg SC every 28 days	Danazol 800 mg/day	Severity scores decreased in both groups; no significant efficacy difference.	Hot flushes were more common with leuprorelin; danazol produced more androgenic/metabolic adverse effects.	
	[60]	Depot leuprolide 3.75 mg monthly	Danazol	Similar efficacy in reducing disease extent and pain/tenderness.	Both regimens were clinically effective, with differing adverse-effect profiles.	Key direct comparison between leuprolide and danazol.
	[61]	Depot leuprolide 3.75 mg/month, IM	Placebo	Physician-rated scores improved for dysmenorrhea, pelvic pain, and pelvic tenderness.	Supports empiric treatment in women with clinically suspected endometriosis; hypoestrogenic symptoms require clinical monitoring.	Clinically relevant empiric-treatment study.
Leuprolide with add-back therapy	[62]	Leuprolide acetate depot 3.75 mg IM every 4 weeks, alone or with add-back	Placebo add-back vs. norethindrone acetate 5 mg/day ± conjugated equine estrogens	Pelvic pain improved significantly in all groups by week 8.	Bone mineral density decreased by 6.3% in the leuprolide-alone arm but was maintained in the add-back groups.	Landmark add-back therapy study.
	[63]	Prior leuprolide ± add-back regimens	Previous randomized arms	Symptoms and examination scores remained improved for at least 8 months after treatment.	Better bone recovery and sustained tolerability were observed in patients who had received add-back therapy.	Long-term follow-up of add-back strategy.
Goserelin	[64]	Goserelin depot 3.6 mg SC every 4 weeks	None	Subjective scores decreased by 86%; 31.5% showed complete disappearance of visible deposits.	Hypoestrogenic adverse effects were observed as expected with ovarian suppression.	Because of its single-arm design, this study is more informative as an early efficacy and safety report than as a comparative trial.
	[65]	Goserelin 3.6 mg every 28 days, SC	Danazol 400 mg/day	Revised AFS scores declined by 53% with goserelin vs. 33% with danazol; symptoms improved in both groups.	Hypoestrogenic effects were more common with goserelin; androgenic effects predominated with danazol.	Comparative efficacy and tolerability study.
	[66]	Depot goserelin acetate 3.6 mg SC every 4 weeks	Danazol 600 mg/day	Similar reductions in endometriosis scores and pain outcomes were reported.	Treatment intolerance was noteworthy, particularly in the danazol group.	Additional comparative clinical trial.
Goserelin versus oral contraceptive/post-surgical strategy	[42]	Goserelin 3.6 mg monthly, SC	Low-dose cyclic oral contraceptive	Pelvic pain and dyspareunia improved in both groups; dysmenorrhea improved significantly in the oral contraceptive group.	Symptoms recurred in most participants after treatment withdrawal.	Illustrates recurrence after treatment cessation.
	[67]	Goserelin + anastrozole	Goserelin alone	Recurrence occurred in 7.5% of the combined-treatment group vs. 35% of the goserelin-alone group; median time to recurrence was longer in the combined group.	Combined blockade prolonged the pain-free interval but was associated with greater short-term bone loss.	Relevant for recurrence-risk discussion.

For clarity, safety findings and interpretative clinical comments were separated in the revised table to improve consistency and comparability across studies.

**Table 3:** Studies evaluating oral GnRH antagonists for endometriosis-associated pain.

Ref	Intervention	Comparator	Duration/Follow-Up	Main Findings	Safety Outcomes	Comments
[77]	Relugolix 10, 20, or 40 mg orally once daily	Placebo; Leuprolide 3.75 mg monthly SC	12 weeks	Relugolix produced a dose-dependent reduction in endometriosis-associated pain. Mean changes in pelvic pain VAS were −3.8 mm with placebo, −6.2 mm, −8.1 mm, and −10.4 mm with relugolix 10, 20, and 40 mg, respectively, compared with −10.6 mm with leuprorelin	Safety findings were consistent with dose-dependent ovarian suppression.	The 40 mg regimen achieved efficacy similar to that observed with leuprorelin.
[75]	Relugolix 10, 20, or 40 mg orally once daily	Placebo; Leuprolide 3.75 mg SC every 4 weeks	24 weeks	Dose-dependent pain reduction with relugolix was maintained through 24 weeks. At the end of treatment, mean pelvic pain VAS changes were −3.2 mm with placebo, −6.8 mm, −9.0 mm, and −11.9 mm with relugolix 10, 20, and 40 mg, respectively, versus −12.7 mm with leuprorelin.	Bone mineral density decline was dose-dependent and, at the 40 mg dose, was comparable to that seen with leuprorelin.	Supports dose–response interpretation.
[78]	Relugolix 40 mg orally once daily	Leuprolide 3.75 or 1.88 mg SC every 4 weeks	24 weeks	Relugolix demonstrated non-inferiority to leuprorelin for reduction in endometriosis-associated pelvic pain. The change in maximum pelvic pain VAS score was −52.6 ± 1.3 with relugolix and −57.5 ± 1.4 with leuprorelin (reported on the original study scale).	Menstruation resumed earlier after treatment discontinuation in patients receiving relugolix.	
[76]	Relugolix 40 mg + estradiol 1 mg + norethindrone acetate 0.5 mg once daily	Placebo	24 weeks	The dysmenorrhea responder rate was 75% (158/212) with relugolix combination therapy compared with 27% (57/212) with placebo. The responder rate for non-menstrual pelvic pain was 58% (124/212) versus 40% (84/212).	Combination therapy was designed to improve tolerability while limiting hypoestrogenic toxicity.	Phase 3 pivotal trial.
[73]	Relugolix 40 mg + estradiol 1 mg + norethindrone acetate 0.5 mg once daily	Placebo	24 weeks	The dysmenorrhea responder rate reached 75% (155/206) with relugolix combination therapy, compared with 30% (62/204) with placebo. For non-menstrual pelvic pain, the corresponding responder rates were 66% (136/206) and 43% (87/204).	Safety profile was consistent with fixed-dose combination treatment.	Phase 3 pivotal trial.
[74]	Relugolix combination therapy	½-	Up to 104 weeks	Improvement in dysmenorrhea, non-menstrual pelvic pain, dyspareunia, and function was maintained through Week 104. At Week 104, responder rates were 84.8% for dysmenorrhea and 75.8% for non-menstrual pelvic pain.	Bone mineral density decreased by less than 1% initially and subsequently remained stable	Long-term extension data.
[72]	Elagolix 200 mg BID + estradiol 1 mg/norethindrone acetate 0.5 mg once daily	Placebo	12 months	At Month 6, the dysmenorrhea response rate was 62.8% versus 23.7%, while the response rate for non-menstrual pelvic pain was 51.3% versus 36.8% compared with placebo. These benefits remained significant through Month 12.	With add-back therapy, bone mineral density change remained below 1% from baseline.	Clinically important for longer-term tolerability.
[68]	Elagolix 150 mg once daily or 200 mg BID	Placebo	6 months	Both elagolix doses improved dysmenorrhea and non-menstrual pelvic pain compared with placebo. Dysmenorrhea response rates at Month 3 ranged from 46.4% to 75.8% for elagolix, versus 19.6% to 22.7% for placebo.	Higher doses were associated with greater hypoestrogenic burden.	
[70]	Elagolix 150 mg once daily or 200 mg BID	-	12 continuous months	Long-term treatment with elagolix was associated with sustained reductions in dysmenorrhea, non-menstrual pelvic pain, and dyspareunia.	After 12 months, dysmenorrhea responder rates reached up to 78.2% with the 200 mg BID regimen.	Long-term extension study.
[79]	Linzagolix 75 mg daily	Placebo	Up to 6 months	Linzagolix 75 mg improved dysmenorrhea (44.0% vs. 23.5%) but did not significantly improve non-menstrual pelvic pain.	Bone loss remained below 1% in the regimen as reported.	Separate lower-dose regimen without conflating outcomes from higher-dose/add-back study.
[80]	Linzagolix 75 mg daily or 200 mg daily + add-back therapy	Placebo	Up to 6 months	At Month 3, linzagolix 200 mg + add-back significantly improved dysmenorrhea (72.9% vs. 23.5%) and non-menstrual pelvic pain (47.3% vs. 30.9%).	Bone loss remained below 1% with the regimen reported.	Separate higher-dose/add-back regimen.

VAS values are reported according to the original scale used in each trial; units and change metrics were clarified in the revised table wherever possible to improve comparability.

## Data Availability

No new data were created or analyzed in this study. Data sharing is not applicable to this article.

## Abbreviations

The following abbreviations are used in this manuscript:

AFS	American Fertility Society
BID	Twice daily
BMD	Bone mineral density
CCL2	C-C motif chemokine ligand 2
COX-2	Cyclooxygenase-2
CXCL8	C-X-C motif chemokine ligand 8
EHP-30	Endometriosis Health Profile-30
GnRH	Gonadotropin-releasing hormone
IL-1	Interleukin-1
IL-1β	Interleukin-1 beta
IL-6	Interleukin-6
IL-8	Interleukin-8
IL-17	Interleukin-17
IM	Intramuscular
JAK/STAT	Janus kinase/signal transducer and activator of transcription
MCP-1	Monocyte chemoattractant protein-1
NGF	Nerve growth factor
NR	Not reported
OCPs	Oral contraceptives
PGE2	Prostaglandin E2
QoL	Quality of life
SC	Subcutaneous
TGF-β1	Transforming growth factor beta 1
Th17	T helper 17
TID	Three times daily
TNF-α	Tumor necrosis factor alpha
TRPV1	Transient receptor potential vanilloid 1
VAS	Visual analogue scale
VEGF	Vascular endothelial growth factor
VEGF-A	Vascular endothelial growth factor A
